# Turning the spotlight on the oligosaccharide chain of GM1 ganglioside

**DOI:** 10.1007/s10719-021-09974-y

**Published:** 2021-02-23

**Authors:** Elena Chiricozzi, Erika Di Biase, Giulia Lunghi, Maria Fazzari, Nicoletta Loberto, Massimo Aureli, Laura Mauri, Sandro Sonnino

**Affiliations:** Department of Medical Biotechnology and Translational Medicine, University of Milano, Via Fratelli Cervi 93, Segrate, Milano 20054 Italy

**Keywords:** GM1 oligosaccharide, Neurodifferentiation, Neuroprotection, Neurodegeneration, Parkinson’s disease, Drug development

## Abstract

It is well over a century that glycosphingolipids are matter of interest in different fields of research. The hydrophilic oligosaccharide and the lipid moiety, the ceramide, both or separately have been considered in different moments as the crucial portion of the molecule, responsible for the role played by the glycosphingolipids associated to the plasma-membranes or to any other subcellular fraction. Glycosphingolipids are a family of compounds characterized by thousands of structures differing in both the oligosaccharide and the ceramide moieties, but among them, the nervous system monosialylated glycosphingolipid GM1, belonging to the group of gangliosides, has gained particular attention by a multitude of Scientists. In recent years, a series of studies have been conducted on the functional roles played by the hydrophilic part of GM1, its oligosaccharide, that we have named “OligoGM1”. These studies allowed to shed new light on the mechanisms underlying the properties of GM1 defining the role of the OligoGM1 in determining precise interactions with membrane proteins instrumental for the neuronal functions, leaving to the ceramide the role of correctly positioning the GM1 in the membrane crucial for the oligosaccharide-protein interactions. In this review we aim to report the recent studies on the cascade of events modulated by OligoGM1, as the bioactive portion of GM1, to support neuronal differentiation and trophism together with preclinical studies on its potential to modify the progression of Parkinson’s disease.

## Introduction

Human gangliosides are glycosylated sphingolipids characterized by the presence of sialic acid. The latter is the general name referring to all the derivatives of neuraminic acid; among them, *N*-acetylneuraminic acid and 9-*O* acetylated *N*-acetylneuraminic acid have been found in humans, where the first one represent most abundant specie [1, 2]. The ganglioside structure is characterized by two distinct portions with different physico-chemical features [3]. They are composed by the highly hydrophobic ceramide, corresponding to an acylated long chain base, called sphingosine, and by the oligosaccharide chain which rather is heavily hydrophilic because of the presence of one or more negatively charged sialic acid. This makes gangliosides amphiphilic compounds capable to establish both hydrophobic and hydrophilic interactions. These interactions have been described in detail and have specific roles in determining several cell properties. The ceramide tail can establish hydrophobic bonds with other membrane lipids and allows the ganglioside to remain firmly anchored to the cell surface. In addition, the ceramide released by plasma membrane associated glycohydrolases can participate to the process of cell apoptosis [4]. In parallel, the oligosaccharide head can interact through mild hydrophilic bonds, i.e. hydrogen bonds, with neighboring membrane components or extracellular ligands.

The ganglioside family is particularly abundant in the nervous system [5–8]. Within it, the ganglioside GM1 has been protagonist of an increasing number of studies [9–11]. It is present within the central nervous tissue of all mammals covering 10–20 % of the total ganglioside content [6].

**Fig. 1:**
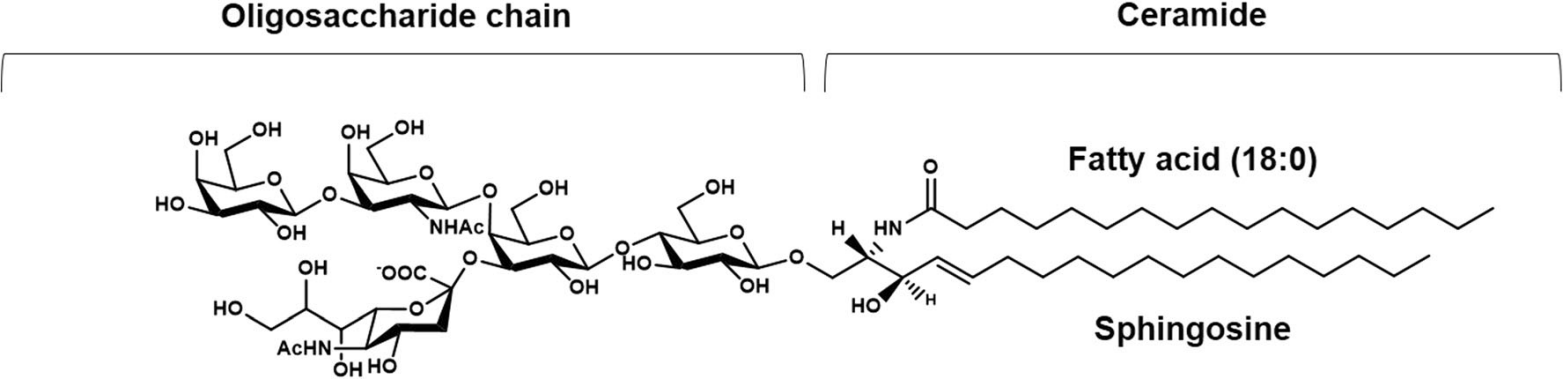
Structure of ganglioside GM1, II^3^Neu5AcGg_4_-Cer

Since the determination of its structure in 1963 [9, 12] the ganglioside GM1 has gained considerable scientific interest for its role in many physiological and pathological processes. The GM1 structure is β-Gal-(1–3)-β-GalNAc-(1–4)-[α-Neu5Ac-(2–3)-]β-Gal-(1–4)-β-Glc-(1–1)-Ceramide, as reported in Fig. 1.

Its neutral tetrasaccharide corresponds to the ganglio series, Gg_4_. The name of ganglioside GM1 was designed by Svennerholm [13], where the capital letter G stands for ganglioside, the following M for monosialylate and the number 1 at the end indicates the specific tetrasaccharide sequence Gg_4_, i.e. β-Gal-(1–3)-β-GalNAc-(1–4)-β-Gal-(1–4)-β-Glc. According to the IUPAC-IUB nomenclature [14], it is named as II^3^Neu5Ac-Gg_4_Cer, where the Roman number indicates the position of the sugar residue to which the sialic acid is linked considering the glucose in first position and the apical Arabic number stands for the linkage position. Like all the nervous system gangliosides, the ceramide portion of GM1 comprises sphingosine with 18 or 20 carbons, which is connected by an amide linkage to a fatty acid, represented by stearic acid in over 80 % of cases in the brain [15, 16]. For major information on GM1 please refer to references [9–11].

GM1 is mainly located into the neuronal plasma membrane, distributed over large portions of the neuron including the axonal, dendritic, and perikaryal membranes [17, 18] as well as at intracellular loci (i.e. neuronal membrane) [19]. As reported by several authors GM1 seems to be largely present at the pre- and post-synaptic terminals where it has been claimed to mediate a series of processes encompassing neuronal differentiation, maintenance of neuronal integrity, synaptic plasticity and repair of cell damage [5, 10, 11, 20–22].

The discovery of the neurotrophic properties of GM1 together with ever more in-depth studies on its molecular structure and dynamics have immediately prompted researchers to investigate the mechanism underlying its properties.

A set of advanced methodologies allowed to disclose that GM1 is enriched in the plasma membrane lipid rafts together with cholesterol, other glycosphingolipids, sphingomyelin, dipalmitoylphosphatidylcholine and a few membrane proteins [9, 10]. The increase in GM1 within lipid rafts, obtained by changes in the activity of the plasma membrane associated sialidase, or by its exogenous administration, correlates with the modulation of the activity of different molecular partners within the cell membrane, such as adhesion receptors, calcium channels and neurotrophin’s receptors belonging to the Trk family [9, 11].

These evidences support a possible direct interaction between GM1 and plasma membrane proteins, but they do not necessarily provide information on the GM1 structural portion responsible for the deriving biological properties: are they due to the hydrophobic component which, in the lipid environment, can alter the characteristics of the membrane and impact on signal transduction mechanisms, or do they depend on the oligosaccharide chain, which protruding into the cellular environment can interact with the exposed domains of membrane receptors?

The ceramide tail is shared by all gangliosides displaying minor structural differences that can modify the membrane fluidity, while the different sugars species with specific composition and configuration of the oligosaccharide chain can make much difference by organizing a network of hydrogen bonds useful for specific molecular interactions.

The possibility that the bioactive properties of GM1 depends only on its oligosaccharide were first suggested by Schengrund and Prouty in 1988, who showed that the administration of the GM1 oligosaccharide (OligoGM1) to S20Y murine neuroblastoma cells in culture induced a neuritogenic effect comparable to that prompted by equimolar administration of the entire GM1 molecule [23]. Unfortunately, this work did not get much feedback from the scientific community and was not pursued further, probably due to the significantly high discussion on gangliosides, that in 80s entered the markets of many countries as drug (first the total ganglioside mixture called Cronassial, and then the pure GM1 called Sygen) for the therapy of peripheral neuropathies [24–27].

30 years later, detailed studies on OligoGM1 have been showing that several fundamental GM1 properties are completely linked to the sole ganglioside head, its oligosaccharide [28–33].

## Strategies to synthesize OligoGM1 and its derivatives

At our best knowledge, currently, the most rapid procedure for obtaining glycolipid-conjugated oligosaccharides starting from the correspondent ganglioside remains the chemical one, through two sequential reactions, established many years ago [34]. The process requires the oxidation of the ganglioside sphingosine double bond with ozone followed by alkaline fragmentation of the parental compound.

**Fig. 2:**
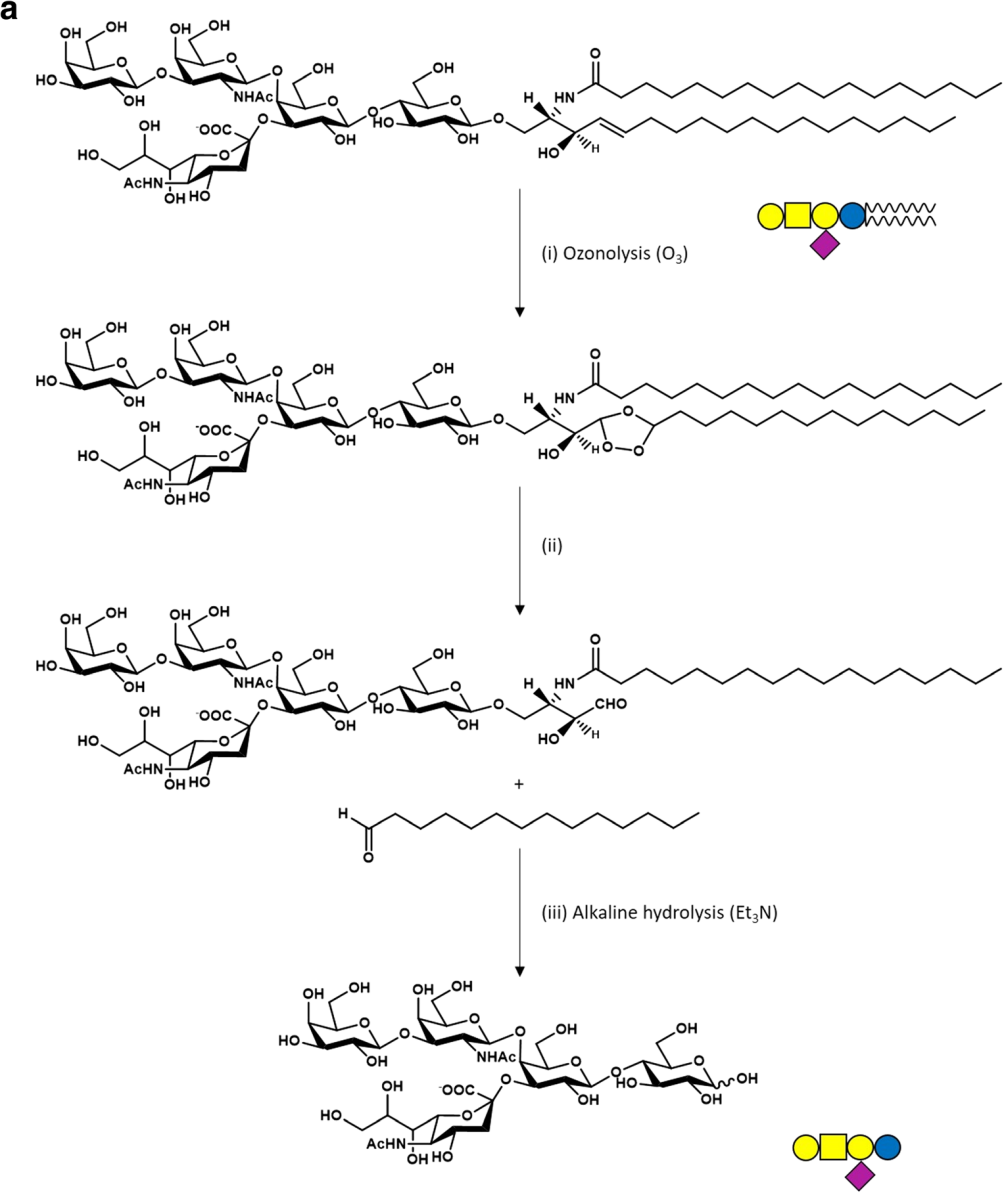

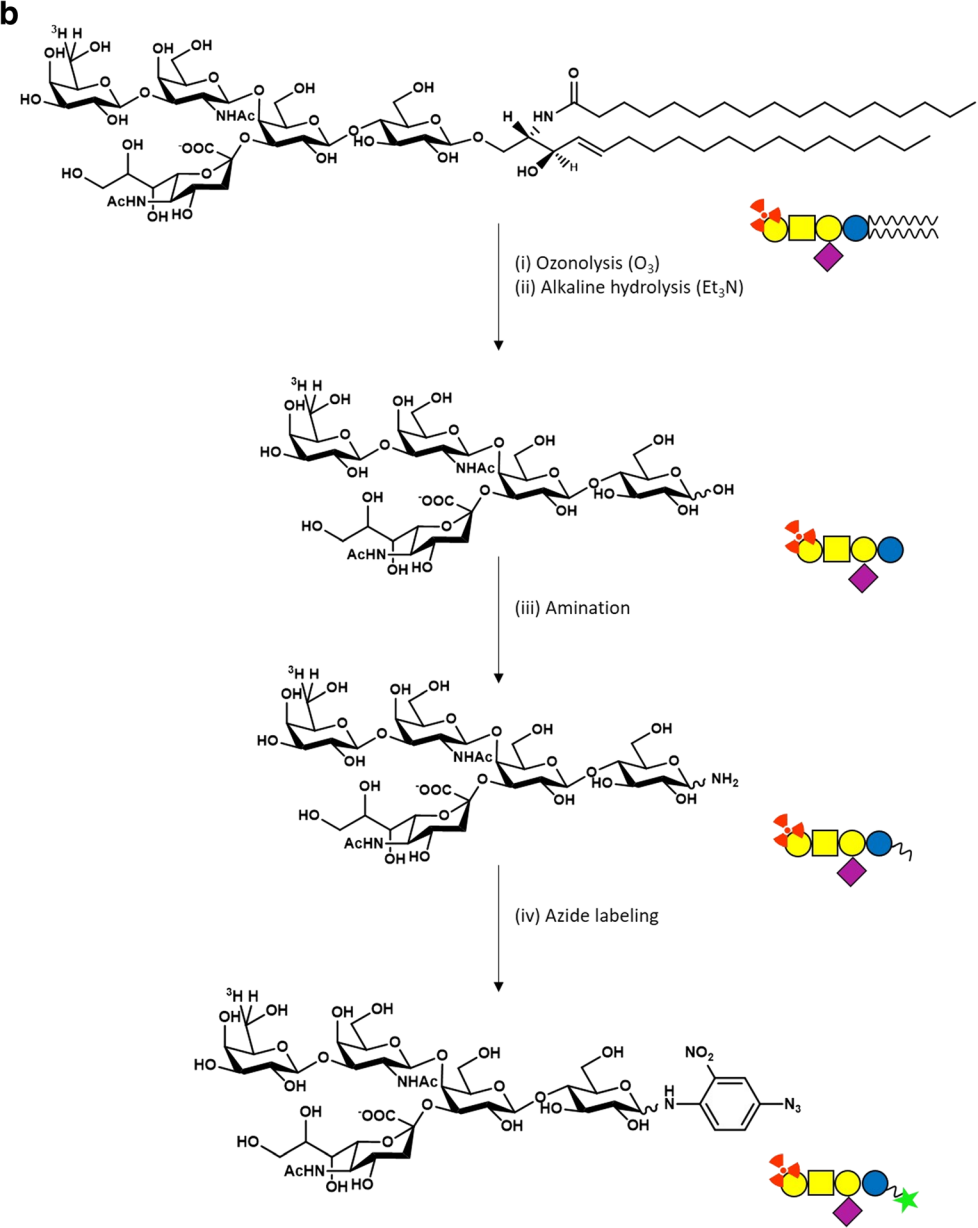

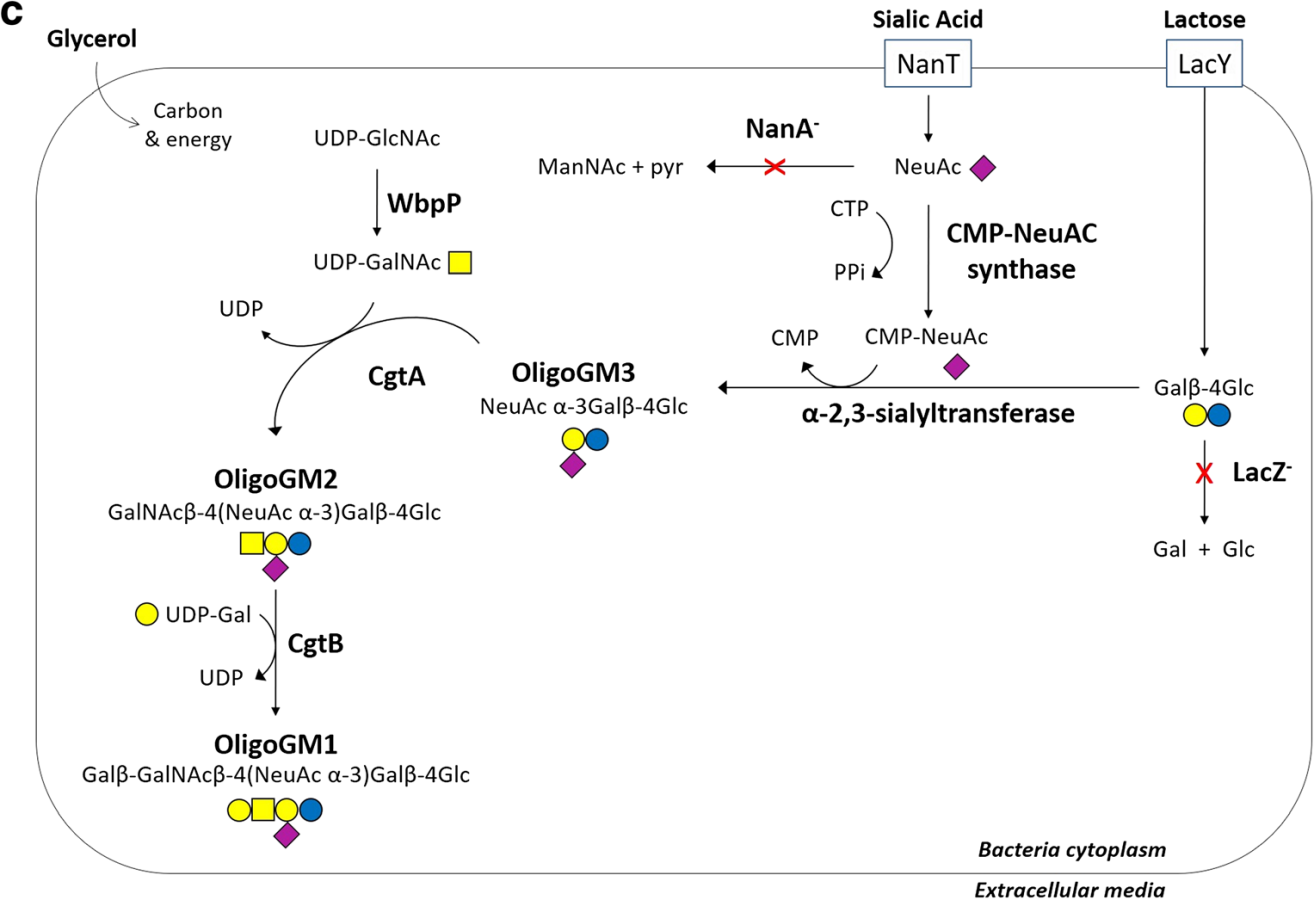
OligoGM1 synthesis strategies. **a** Scheme of the chemical synthesis of OligoGM1. GM1 undergoes to ozonolytic (O_3_) process in methanol (i) generating a desphingosine aldehyde product and a miristic aldehyde (ii), followed by alkaline hydrolysis in triethylamine (Et_3_N) (iii) releasing OlgoGM1; **b** Scheme of the chemical synthesis of tritium-labeled and photoactivable OligoGM1. [^3^H]GM1 undergoes ozonolysis (i) in methanol followed by triethylamine alkaline hydrolysis (ii) releasing [^3^H]OligoGM1. The latter is subjected to the amination process (iii) and the [^3^H]OligoGM1-NH_2_ is azide labeled (iv) with 2-nitro-fluorophenylazide in dimethylformamide (DMF) and tributiylamine (Bu_3_N) in dimethylsulfoxide (DMSO) to obtain [^3^H]OligoGM1-N_3_. **c** OligoGM1 biosynthesis in engineered *Escherichia coli.* Lactose and sialic acid (Neu5Ac) are internalized by the specific permeases LacY and NanT but cannot be degraded because of β-galactosidase (LacZ) and aldolase (NanA) deletion. CMP-Neu5Ac synthase activates Neu5Ac into CMP-Neu5Ac which is transferred onto lactose by α2,3-sialyltransferase (encoded by Lst), to form II^3^αNeu5Ac-Lac (sialyllactose, OligoGM3). The use of the endogenous pool of UDP-GalNAc produced by the recombinant UDP-GlcNAc C4 epimerase (WbpP) allows β1,4-GalNAc transferase (CgtA) to catalyze the glycosylation of sialyllactose to form II^3^αNeu5Ac-Gg_3_ (OligoGM2). This compound is substrate for the β1,3-galactosyltransferase (CgtB) to yield II^3^αNeu5Ac-Gg_4_ (OligoGM1). CTP, cytidine triphosphate; Ppi, inorganic pyrophosphate. Oligosaccharide sugar code is according to Varki et al. [36]

Briefly, GM1 purified from a mixture of gangliosides extracted from mammal brains is solubilized in methanol and slowly saturated with ozone for about 2 hours, a reaction that breaks the double C-C bond in C4 position generating a desphingosine aldehyde product and a miristic aldehyde [35]. After solvent evaporation, the residue is brought to alkaline pH with the addition of a strong base. The original method used NaOH, but now we are used to employ triethylamine, which has the advantage to be removed by evaporation. The pH is kept always higher than 10 and, after 48–96 hours (depending on the starting GM1 concentration) at room temperature (i.e. 20–23°C), the sialo-oligosaccharide is dissociated from the reactive desphingosine compound. After solvent evaporation, under strong vacuum, GM1 oligosaccharide is purified by flash chromatography using chloroform/methanol/2-propanol/water 60/35/5/5 v/v/v/v as eluent. This procedure leads to approximately 50 % of final yield (Fig. 2a).

To obtain a radiolabeled OligoGM1, GM1 containing tritium in position 6 of external galactose is prepared by enzymatic oxidation with galactose oxidase followed by reduction with sodium boro[^3^H]hydrate [37]. The [^3^H]GM1 obtained is then subjected to ozonolysis and chromatographic purification as above reported to obtain the correspondent [^3^H]OligoGM1 and as described in the first two steps reported in Fig. 2b.

Starting from [^3^H]OligoGM1, photoactivable derivatives are prepared to be used in studies aimed to identify molecular interactors. Tritium labeled OligoGM1 modified to carry a photoactivable nitro-phenyl azide group linked to the glucose residue is synthetized as previously reported [28] by dissolving [^3^H]OligoGM1 in 33 % ammonia and then treating with ammonium hydrogen carbonate under stirring for 48 hours at 40°C (Fig. 2b). The crude amino-[^3^H]OligoGM1 is dissolved in dry dimethylformamide (DMF), and following 2-nitro-4-fluoro-phenylazide and tributylamine in dry dimethyl sulfoxide (DMSO) are added under dark condition. Keeping the dark condition for all the process, the reaction mixture is stirred for 16 hours at 80°C. After solvent evaporation, the photoactivable tritium labeled OligoGM1, [^3^H]OligoGM1-N_3_, is purified by flash chromatography using chloroform/methanol/2-propanol/water 60:35:5:5 as eluent [38]. [^3^H]OligoGM1-N_3_ solubilized in methanol is stored at 4°C.

The administration of [^3^H]OligoGM1-N_3_ to the cell culture medium, followed by UV-light illumination, leads to the formation of highly reactive nitrene intermediate which immediately generates a covalent bond with the adjacent molecules. In this way proteins molecules close enough (≤ 4 Å), by binding to the photoactivated tritium labeled oligosaccharide, become radioactive [28, 30, 38–41]. Following cell protein/lipid extraction, the presence of the radioactivity at the level of a specific protein/lipid signal suggests the identification of a possible partner of OligoGM1 [28, 30].

Although the chemical procedure is the most rapid and is an already employed approach, there are reasons that strongly suggest to look for alternative strategies for the production of large amount, i.e. drug quantities, of GM1 oligosaccharide: *(i)* it cannot be excluded that the GM1 animal extraction process can lead to eventual protein contamination and thus to neurological pathologies such as prion disease; *(ii)* the potential use of the OligoGM1 as drug for therapy of neurodegenerative diseases (see below) would require an incredible great quantity of animal brains for the production of tons of GM1, this introducing a problem concerning the removal of wastes as well as ethical controversy. This evokes new biotechnology researches aimed to produce oligosaccharide chains through bacterial engineering. One of the strategies for the bacterial production of the OligoGM1 is summarized in the diagram in Fig. 2c [42–44].

Mutant *Escherichia Coli* strains devoid of β-galactosidase and Neu5Ac aldolase activity are transformed to overexpress the five genes coding the enzymes required for the synthesis of GM1 carbohydrate: CMP-Neu5Ac-synthase, α-2,3-sialyltranferase, UDP-GlcNAc C4 epimerase, the β-1,4-N-acetylgalactosaminyltransferase and the β-1,3-galactosyltransferase. The bacteria are grown with glycerol as carbon and energy source, and supplied with exogenous lactose and Neu5Ac which accumulate in the cytoplasm, where Neu5Ac is converted into CMP-Neu5Ac to be transferred onto lactose and form sialyllactose by α-2,3-sialyltranferase activity. The recombinant UDP-GlcNAc C4 epimerase produces UDP-GalNAc from endogenous UDP-GlcNAc and then the β1,4-GalNAc transferase catalyzes the glycosylation of sialyllactose to form II^3^Neu5Ac-Gg_3_ (OligoGM2). This compound serves as an acceptor for β1,3-galactosyltransferase and reacts to yield II^3^Neu5Ac-Gg_4_ (OligoGM1). Actually, we are working on improving this strategy with excellent results that will be presented soon.

## OligoGM1 biological properties

Studies on the OligoGM1 biological properties have been developed in relation to neuronal differentiation, maturation and protection, explaining some mechanistic aspects on how GM1 is able to regulate multiple cellular processes using the carbohydrate portion, and to initiate signal transduction cascades on the plasma membrane [28–33].

### Neuronal differentiation

**Fig. 3:**
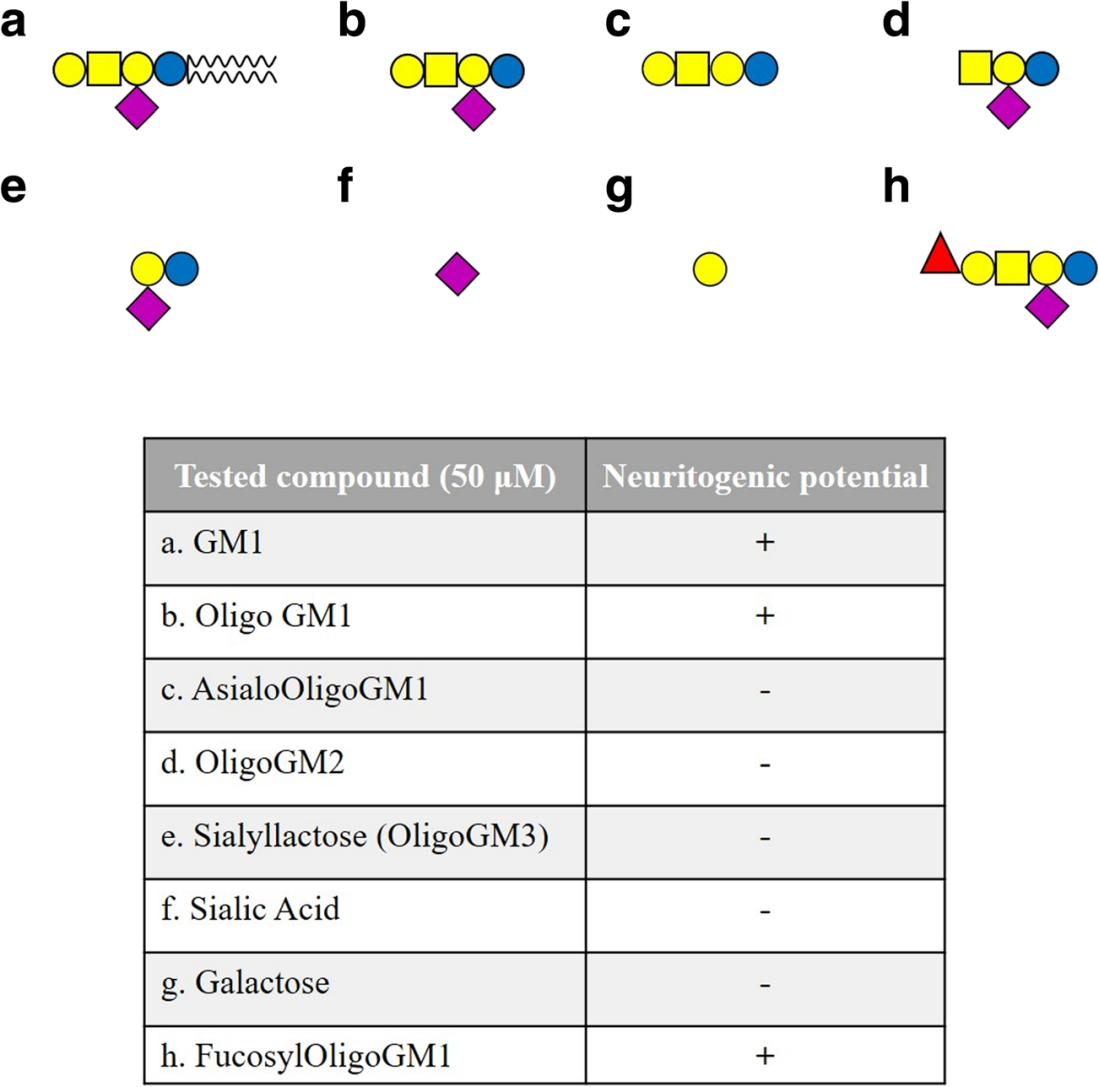
Neuritogenic potential of GM1, OligoGM1 and isolated carbohydrate’s components in N2a cells. **a** The symbolic structure of tested compounds according to [36], whose specific names are reported in the table below. **b** The effectiveness of the compound in inducing neuritic growth is represented by the **+** symbol. The symbol **-** indicates that the compound fails to induce neuritogenesis

OligoGM1 neuritogenic potential was firstly confirmed in Neuro2a (N2a) murine neuroblastoma cells [28, 30]. The addition of OligoGM1 to the N2a culture medium induced the emission of neuritic prolongations and the elevation of the neurofilament proteins analogously to what was observed administering the entire GM1 [28]. Incubations with OligoGM1 derivatives such as asialo-OligoGM1 (Gg_4_), OligoGM2 (II^3^Neu5Ac-Gg_3_), OligoGM3 (sialyl-lactose), or the simple sugars galactose and sialic acid were performed in order to clarify the minimal structure required to promote neurite elongation (Fig. 3). None of these compounds was able to induce any cell morphological changes [28]. Interestingly, the administration of fucosylated OligoGM1 (IV^2^αFucII^3^Neu5Ac-Gg_4_) prepared from fucosylated GM1 was able to induce neurite sprouting in a similar way as GM1 and OligoGM1 did [28]. Recalling that glycohydrolases show low affinity on the ganglioside oligosaccharides we can exclude that the neurogenic effect exerted by Fuc-OligoGM1 derives by its transformation into OligoGM1 at the cell surface [45, 46].

These results suggested that the β-Gal-(1–3)-β-GalNAc-(1–4)-[α-Neu5Ac-(2–3)]-β-Gal-(1–4)-Glc represents the minimal structure required by the saccharide to exert its neuronal function and the addition of an α-fucose at position 2 of the external galactose is irrelevant for the process.

Further studies have been performed by culturing undifferentiated mouse cerebellar granule neurons in the presence of OligoGM1 for the time necessary to cell differentiation [32]. Time-course live cell imaging showed the capability of the pentasaccharide to accelerate neuron maturation process, increasing the aggregation rate with respect to control cells and prompting the expression of specific neuronal protein and lipid markers in the first hours of neuronal culture [32].

These results have highlighted the great neurotrophic potential of OligoGM1, superimposable to that of GM1 previously demonstrated using similar experimental models [47–50].

### Neuronal protection

**Fig. 4:**
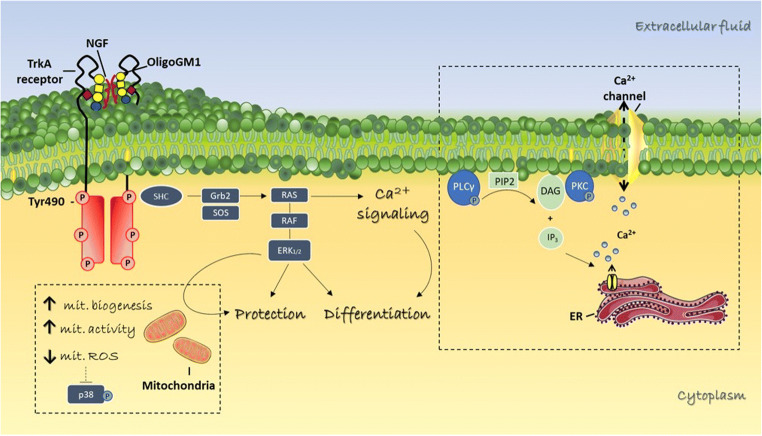
Diagram of the proposed mechanism for OligoGM1 modulatory activities in N2a cells. At cell surface, OligoGM1 is able to directly bind and stabilize the TrkA-NGF complex triggering the hyperphosphorylation of the receptor at Tyr490 and the downstream MAPK pathway. The activated cascade stimulates several cellular events including the calcium signalling, the differentiation in neuron-like cells, the protection against neurotoxic stimuli (i.e. MPTP), the enhancement of mitochondrial bioenergetics. This image is updated from [28, 29, 31, 33]. GM1 saccharide representation is according to [36]. ERK, extracellular signal regulated protein kinases 1 and 2; Grb2, growth factor receptor bound protein 2; Gab1, Grb2-associated binder-1; RAS, GTP-binding protein; RAF, serine/threonine kinase; SHC, transforming protein 1; SOS, son of sevenless; p38 Mitogen-activated protein kinase, PLCγ, Phospholipase Cγ; PIP2, Phosphatidylinositol 4,5-bisphosphate; DAG, Diacylglycerol; IP_3_, Inositol trisphosphate; PKC, Protein kinase C

Several studies showed the neuroprotective and reparative properties of GM1 against neuronal damage, caused by the addition of glutamate or peroxidizing agents such as hydrogen peroxide (H_2_O_2_) or 1-methyl-4-phenyl-1,2,3,6- tetrahydropyridine (MPTP) [51–57]. Therefore, the possibility that the GM1 oligosaccharide could also contribute to explain the GM1 neuroprotective function was investigated. In this context, a 24-hours pretreatment of N2a cells with OligoGM1 prevented the neurotoxic action of MPTP, increasing the cell survival rate [29]. The MPTP is a pro-neurotoxin that, upon cell oxidative conversion into 1-methyl-4-phenylpyridinium (MPP^+^), inhibits the complex I of mitochondria electron transport chain inducing ATP depletion, reactive oxygen species (ROS) production and finally cell death [58–60]. Importantly, OligoGM1 pretreatment reduced mitochondrial ROS production possibly through the modulation of mitochondria quality control pathway by p38 MAPK, preventing oxidative stress and reducing N2a cell death (Fig. 4) [29, 31].

This work suggests not only a probable therapeutic potential of OligoGM1 from a phenomenological point of view, but also from a mechanistic one it had shown that OligoGM1 could influence neuronal mitochondrial activity, somehow enhancing its functionality.

### Mitochondria modulation

N2a cells grown in the presence of OligoGM1 displayed an increased number of mitochondria as revealed by the increase expression of the mitochondrial markers (i.e. Tom20 and HTRA2) as well as increase in mitochondrial DNA [31]. Furthermore, transmission electron microscopy analysis has shown that the mitochondria of N2a cultured in the presence of OligoGM1 appear more electrodense and with an increased number of cristae compared to the controls [31]. These data indicated a higher mitochondrial functionality over the increased mitochondriogenesis, a result confirmed by the evaluation of greater oxygen consumption rate by the cells treated with OligoGM1 together with the increase in ATP production (Fig. 4) [31]. This mitochondrial effect exerted by OligoGM1 could be associated with the induction of cell differentiation previously observed on the same experimental model, which is accompanied by the metabolic change observed following differentiation of tumor cells.

Interestingly, OligoGM1 has been found to induce an increase in mitochondrial functionality and the activity of complexes I and II of the electron transport chain in a mitochondrial impairment model represented by N2a chronically exposed to subtoxic levels of ethidium bromide [31, 61].

## OligoGM1 mode of interaction

From theory it is easily deducible that the way of interaction between cells and the entire GM1 or its saccharide chain is totally different, since their chemical/physical properties are certainly different: GM1 is an amphiphilic molecule forming aggregates in aqueous solutions in equilibrium with about 10^-9^ M monomers, while its saccharide is completely soluble in monomeric form [62–64].

Few hours later from GM1 administration to cells, under defined experimental conditions, the monomeric GM1 is taken up by the cell becoming a component of the plasma membrane external layer, almost indistinguishable from the endogenous ganglioside, so that it can also be internalized reaching the lysosomes where its catabolic fragments are then recycled for the biosynthesis of all sphingolipids [65].

To clarify how the hydrophilic oligosaccharide interacts with the cells, time-course *in vitro* experiments employing an isotopic radiolabeled form of OligoGM1 were conducted. These essays showed that no trace of radioactivity was inside the cells at any time point analyzed [28, 32]. This result allows to conclude that the neurotrophic and protective effect of OligoGM1 on neuronal and neuronal-like cultures is due to an action of the pentasaccharide on the cell surface, i.e. by the interaction with membrane receptor(s). After all, this finding is not surprising considering that the neuritogenic effect exerted by GM1 is observed following a local membrane increase in ganglioside monomers, precisely at the level of the growing axon cone by elicitation of the activation of membrane Trk receptors [48]. Moreover, the increase in endogenous GM1 was also observed in cortical neurons following the application of a neurotoxic stimulus as a possible mechanism of neuronal self-defense [66]. In this way, clustering of GM1 monomers leads to a local enrichment of oligosaccharide chains within the membrane glycocalyx, which could be directly involved in the interaction with membrane receptors, influencing their functionality and downstream pathways.

## OligoGM1 mechanism of action

According with the interaction experiment, OligoGM1 was found to increase the phosphorylation of the nerve growth factor (NGF) specific TrkA receptor on tyrosine 490 associated with the increase in MAP kinases (MAPK) activity when added to N2a cells and primary granule neurons in culture [28, 32]. The neuritogenic and protective effect of OligoGM1 was abolished following the chemical or genetic inhibition of TrkA, confirming that the TrkA-MAPK pathway mediated the biological effects of the saccharide [28, 29]. In primary neurons the OligoGM1-induced TrkA-MAPK activation was associated with the increase in Fak and Src protein phosphorylation, mediators of cell motility and responsible for the increase in neuron clustering observed following the administration of OligoGM1 [32].

### TrkA-NGF-OligoGM1 partnership

The TrkA receptor and GM1 ganglioside partnership has been reported in several papers [49, 53, 67–69] where it was emphasized that the NGF receptor requires the presence of GM1-enriched membrane to be active, while the GM1 absence negatively correlates with TrkA function. Specifically, Mutoh demonstrated that the direct binding of GM1 to Trk enhance the effects of NGF on Trk kinase activity [70].

Accordingly information about TrkA receptor as a protein containing a glycosphingolipid binding domain capable to establish a stable complex with GM1 has been already reported [71], but how exactly the glycolipid-protein interaction occurred on the cell membrane had not been clarified. This aspect was elucidated by experiments carried out with the radioactive and photoactivable derivative of OligoGM1 (^3^H-OligoGM1-N_3_) [28]. After the administration of this derivative to the N2a culture, the cells were irradiated with UV light. The proteins were then extracted, separated by SDS-PAGE and transferred onto a PVDF membrane. The membrane was subjected to autoradiography and subsequently incubated with a TrkA specific antibody. The overlapping between the radiolabeled band and TrkA specific immunosignal was observed, indicating a direct interaction between the GM1 oligosaccharide and the extracellular domain of TrkA receptor. This discovery demonstrated for the first time a direct association between the NGF receptor and one specific portion of GM1, i.e. the oligosaccharide. In addition, we ruled out the involvement of ceramide in the GM1-TrkA interaction by preparing and employing two modified forms of radiolabeled and photoactivable GM1: one carrying a photoactivatable phenylazide group associated with the oligosaccharide chain, and the other one carrying the same group within the hydrophobic portion [30]. Following cell administration and UV irradiation, the analysis of protein pattern led to the identification of a radiolabeled band overlapping the TrkA immunoreactive band only by using the ^3^H-GM1 derivative containing the photoactivatable group conjugated to the oligosaccharide head. This finding clearly indicated that the interaction between TrkA and ganglioside GM1 occurred outside the cell at the level of the oligosaccharide. Interestingly, we never detect TrkA within plasma membrane microdomains, where GM1 is enriched. These data suggest that the interaction between the two molecules does not involve a receptor shift into the lipid rafts and it is therefore probably made possible by a flopping down of the extracellular domain of TrkA towards the oligosaccharide chain of GM1.

**Fig. 5:**
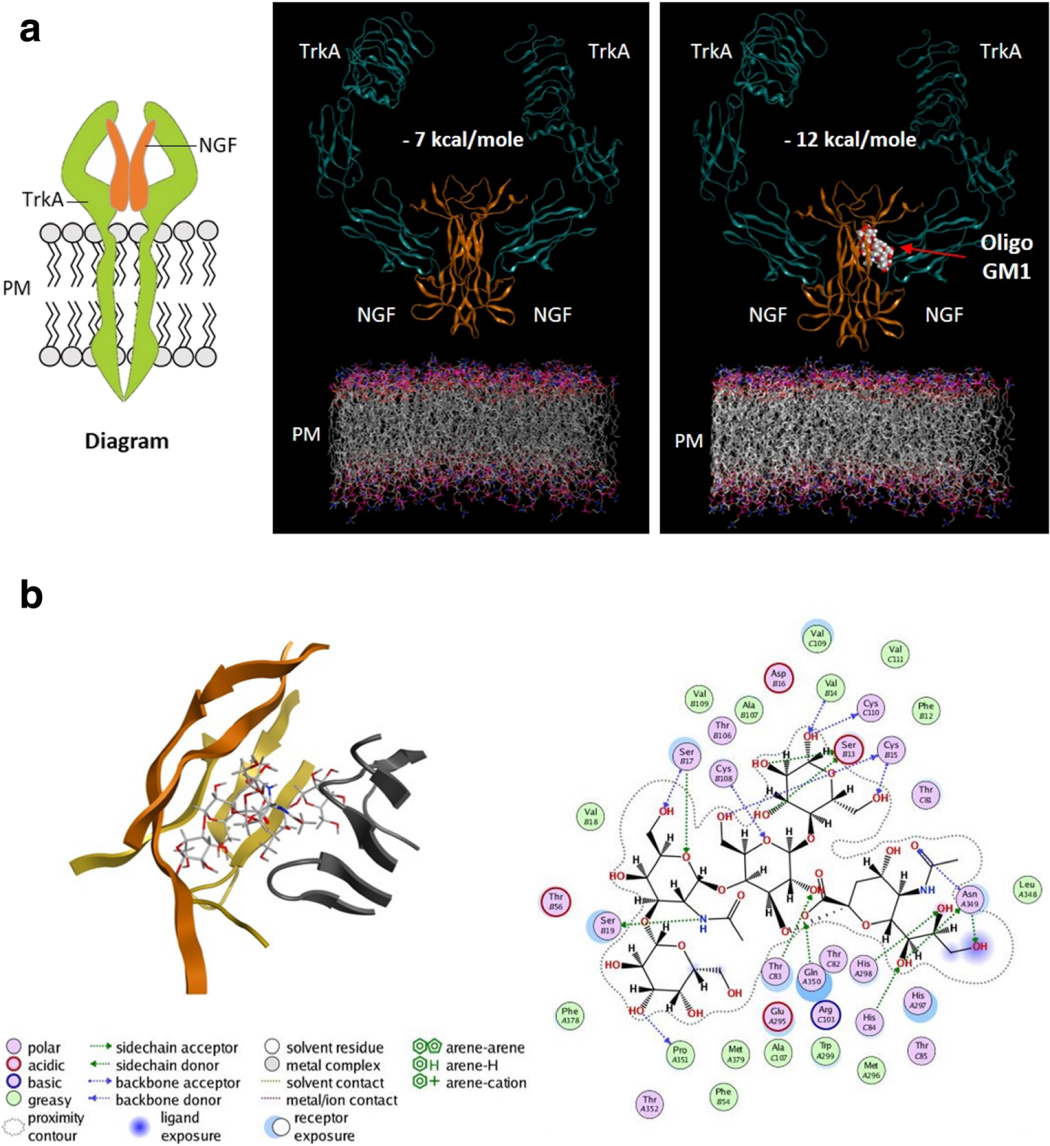
Molecular docking analyses of TrkA-NGF-OligoGM1 complex. **a** Diagram depicting the TrkA-NGF complex at cell surface and representation showing the TrkA-NGF interaction in absence (left) or in presence (right) of OligoGM1 as crystallographic complex. The binding energy associated to the TrkA-NGF complex is reduced from − 7 kcal/mol to -12 kcal/mol when OligoGM1 is present into the complex TrkA receptor is represented in magenta ribbons; NGF molecules in orange ribbons; OligoGM1 using space-filling model. **b** Representation of bonds between the extracellular residues of TrkA, NGF and OligoGM1

Furthermore, molecular docking analyses confirmed that the extracellular portion of TrkA presents a specific binding domain for GM1 saccharide chain (Fig. 5) [28]. Indeed, analyzing the crystal of the TrkA-NGF complex there seems to be a pocket available to host a possible other ligand. *In silico* experiments revealed that the GM1 oligosaccharide fits perfectly into this pocket. Additionally, when GM1 oligosaccharide binds the TrkA-NGF complex the free energy of association is significantly reduced, suggesting that OligoGM1 acts as a bridge able to increase and stabilize the TrkA-NGF molecular interactions. In fact, a high number of weak bonds between specific TrkA extracellular residues, NGF and OligoGM1 have been identified as reported in Fig. 5b. The pocket represented in the crystallized structure has two holes between the TrkA and the NGF: one facing the plasma membrane and the other the extracellular space. Likely, the hole facing the plasma membrane represents the passage for the ceramide tail, while the one in the extracellular space allows the presence of other sugars in addition to those of GM1 pentasaccharide, as in the case of Fucosyl-OligoGM1.

The literature cited above firmly highlights that GM1 oligosaccharide stimulates the activation of TrkA receptor by enhancing the binding with it natural ligand, the NGF. Indeed, all the *in vitro* and *in vivo* experiments have been conducted in the presence of NGF naturally present in the serum of the culture media as well as physiologically secreted in animal body. However, we cannot exclude that sole OligoGM1 interaction is sufficient to modulate the TrkA function. For this purpose, we are working at different levels to define the essentiality of NGF presence in the GM1 (GM1 oligosaccharide) TrkA mediated pathway.

## Network of intracellular pathway modulated by OligoGM1

Intracellular calcium levels increased in few minutes after OligoGM1 administration to the N2a culture medium, as measured by live cell calcium imaging [33]. Interestingly, the selective inhibition of TrkA kinase activity, resulted in no change in cell calcium levels, indicating that the activation of TrkA constitutes an upstream event over the opening of calcium ion channels [33]. TrkA activation is followed by the activation of the PLCγ phospholipase which converts phosphatidylinositol 4,5-bisphosphate (PIP2) to inositol 1,4,5-trisphosphate (IP3) and diacyglycerol (DAG) [72] (Fig. 4). The diacyglycerol (DAG) recruits Protein kinase C (PKC) to the membrane and it has been observed that the OligoGM1 induces an increased PKC membrane localization, precisely at the level of the lipid rafts, together with a higher degree of protein phosphorylation [73–76]. IP3 binds specific receptors on the endoplasmic reticulum to release luminal calcium storage into the cytoplasm [75]. Pretreatment of N2a with a specific IP3 receptor inhibitor reduces the increase in intracellular calcium induced by OligoGM1 but does not completely abolish its effect, suggesting that the calcium flows moved by the glycan come not only from intracellular storages (endoplasmic reticulum) but probably also from the extracellular environment [33]. In fact, by adding extracellular or intracellular calcium chelating agents (ethylene glycol tetraacetic acid (EGTA) or 1,2-bis(o-aminophenoxy)ethane-N,N,N′,N′-tetraacetic acid (BAPTA) respectively) to the culture medium, the calcium dependent neuritogenic effect induced by OligoGM1 was abolished [33].

In the past, GM1 ganglioside had been found to regulate intracellular calcium influx by modulating the opening of low-threshold voltage-dependent T-type calcium channels and metabotropic transient receptor potential channel 5 (TRPC5) in neuroblastoma cells and primary neurons [77–80]. The possibility that these channels could be under the modulating control of OligoGM1 still needs to be investigated.

Calcium is a second messenger that regulates various cellular processes encompassing cell differentiation, emission of neuritic extensions, neuronal electrical activity and protection from noxious stimuli. The detection of changes in intracellular calcium level following the administration of OligoGM1 is consistent with the observed neurotrophic and protective effects. In agreement, proteomic analysis has shown that N2a treated with OligoGM1 express exclusive protein sets, among which about 13 % are involved in the signaling of calcium, such as cell adhesion, cell cycle regulation and endocytic transport proteins [29]. The neuronal calcium sensor 1 (NCS1) involved in the calcium sensing was also exclusively expressed in N2a treated with OligoGM1 [29].

The proteomics associated with the gene ontology analysis showed that OligoGM1 induced the expression and exclusive upregulation of proteins involved in the processes of cell migration, neuritogenesis and mitochondrial functionality, confirming the phenomenological experimental evidence on the ability of the oligosaccharide to orchestrate these cellular events [29] (Table 1).

**Table 1:** List of selected cellular event prompted by OligoGM1 administration to N2a cells

Cellular event	Oligo-ONLY protein cluster	DAVID cluster	
Activation of calcium channel	Calcium-dependent Cadherin-like proteins		7
	Calmodulin-binding proteins		14
	EGF-like calcium-binding proteins		35
	Ion channels		52
Cell migration, clusterization, adhesion	Laminin G-like proteins		5
	Calcium-dependent Cadherin-like proteins		7
	Tyrosine-protein kinases involved in the regulation of cell adhesion, membrane raft, and positive regulation cell migration		21
	Proteins mediating the extracellular matrix organization		27
	EGF-like domain proteins involved in extracellular matrix growth		35
	Calcium-dependent Cadherin-like proteins modulating cell-cell adhesion		7
	Transmembrane glycoproteins		47
Suppression of proinflammatory molecules and microglial activation	Mitochondria-related proteins		4
	Peroxisome-related proteins		31
	Proteins involved in fatty acid metabolism and modulating PPAR signaling pathway (anti-inflammation, mitochondrial biogenesis)		6
	Proteins involved in PI3K/AKT signaling pathway		16, 34
Neuroprotective effect	Proteins regulating NO signaling Pathway		10
	Mitochondria-related proteins		4
	Proteins involved in fatty acid metabolism and modulating PPAR signaling pathway (anti-inflammation, mitochondrial biogenesis)		6
	Proteins involved in PI3K/AKT signaling pathway		16, 34
	Proteins mediating MAPK signaling pathway, Wnt signaling pathway and maintaining dopaminergic synapses		20
	Proteins involved in tyrosine-protein kinase signaling, MAPK cascade		21
	Small GTPase (Ras, MAPK) mediating signal transduction		36
Immunity modulation	VIP and PACPA neuropeptides which inhibit the apoptosis of activated T cell by blocking NO production		10

The bioinformatic analysis was performed using DAVID software on proteins exclusively expressed in OligoGM1-treated cells comparing OligoGM1 vs. CONTROL N2a. For full list of proteins see [29]

Importantly, the proteomic analysis has highlighted the OligoGM1 influence on inflammation and immune system, already partially known for GM1, but only at the beginning of our understanding for OligoGM1 [29]. For a complete list of proteins expressed in N2a incubated with OligoGM1 refer to the work of Chiricozzi et al. 2019 [29].

## OligoGM1 as a drug for neurodegenerative diseases

With their saccharide head, gangliosides contribute significantly to the cell surface glycans in neuronal cells. Recognizing the oligosaccharide chain of ganglioside GM1 as an essential biosignaling molecule suggests to focus the attention on the composition balance of the cell glycocalyx, in which even small variations of carbohydrate chains could provoke responses that determine cell functioning and fate. On the other hand, alterations in the glycoconjugate composition of the membrane could determine or contribute to the pathogenesis of various neurological diseases [81–84]. In dementias and neurodegenerative diseases, reduction in the content of gangliosides and in particular of GD1a and GM1 have been found in injured brain areas of affected patients compared to normal subjects of the same age [5, 83, 85–87].

The pioneering work of Svennerholm published in 1994 reports that the total content of human gangliosides remains unchanged from the age of 20 to 70 years, after which a progressive decline is appreciated [6, 86]. The ganglioside pattern showed continuous change with aging, with decreasing proportions of GM1 and its metabolic reservoir GD1a (converted to GM1 by membrane-localized sialidase) and increasing proportions of GD1b, GM3, and GD3 [6]. Therefore, it follows that in neurodegenerative diseases an acceleration of the ganglioside decline with respect to the “physiological” aging-dependent one occurs. This is especially true in Parkinson’s disease (PD) where a significant GM1 deficiency has been found in patients’ striatum and *substantia nigra*, along with a reduction of the glycosyltransferases necessary for the ganglioside biosynthesis [85, 87, 88]. Thus, according to the concept that GM1 has a neurotrophic and neuroprotective role, the hypothesis that the reduction of GM1 level below a certain physiological threshold could lead to insufficient neuronal trophic support and promote a neurodegenerative process becomes consistent. This seems to be confirmed by the features of the mouse model where the homozygous or heterozygous deletion of the *B4GalNT1* gene (*B4GalNT1*^*-/-*^, *B4GalNT1*^*+/-*^) coding for the enzyme β-1,4 *N*-acetylgalactosaminyltransferase 1, necessary for the biosynthesis of a series gangliosides, including GM1, leads middle age mice to develop the anomalies typical of human PD: gait defects, catalepsy and loss of forelimbs strength, accompanied by pathognomonic neurological lesions represented by the loss of nigrostriatal dopaminergic neurons, reduction of dopamine in the striatum and aggregation and brain accumulation of α-synuclein (α-syn) protein. Moreover, these mice also display non-motor symptoms characteristic of PD affecting the gastrointestinal, sympathetic, cardiac, and cerebral cognitive systems [87, 89, 90].

Systemic administration of GM1 to the heterozygous mice did not get promising results, due to the strong amphiphilic properties of GM1 that prevent to cross the blood brain barrier (BBB) [87, 91]. Past studies showed that a very scant quantity of GM1 intraperitoneally injected into mice reached the brain, a part of which was found in the brain blood vessels [92]. The reduced capability of GM1 to cross the BBB has been recently confirmed in primates subjected to PET analysis after intravenous injection of fluorinated [^18^F]GM1 [93]. This study demonstrated that only the 0.4 % of injected GM1 entered the brain. At least a part of this GM1 is not in blood vessels and thus seems associated to neurons.

**Fig. 6:**
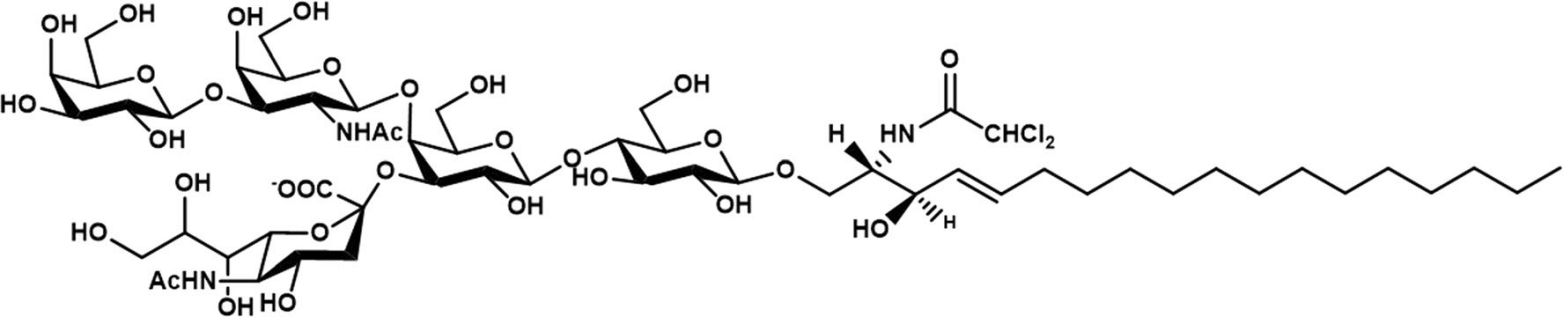
Structure of LIGA20

But, interestingly, the intraperitoneal administration of the membrane-permeable analogue of GM1, LIGA20, was found to improve the motor phenotype of transgenic mice and to reduce the mortality of dopaminergic neurons and the amount of α-syn aggregates, as well as the original GM1 [87]. LIGA20 was developed with the aim to have a more hydrophobic compound capable to cross more efficiently the BBB with respect to GM1 and, for this purpose, it is modified in the hydrophobic portion where the stearyl group is replaced with a dichloroacetyl group but it maintains the structure of GM1 oligosaccharide unaltered (Fig. 6).

Importantly this work suggested that the hydrophobic portion of GM1 was not indispensable while the carbohydrate was the essential one for the physiological maintenance of dopaminergic neurons *in vivo* too.

**Fig. 7:**
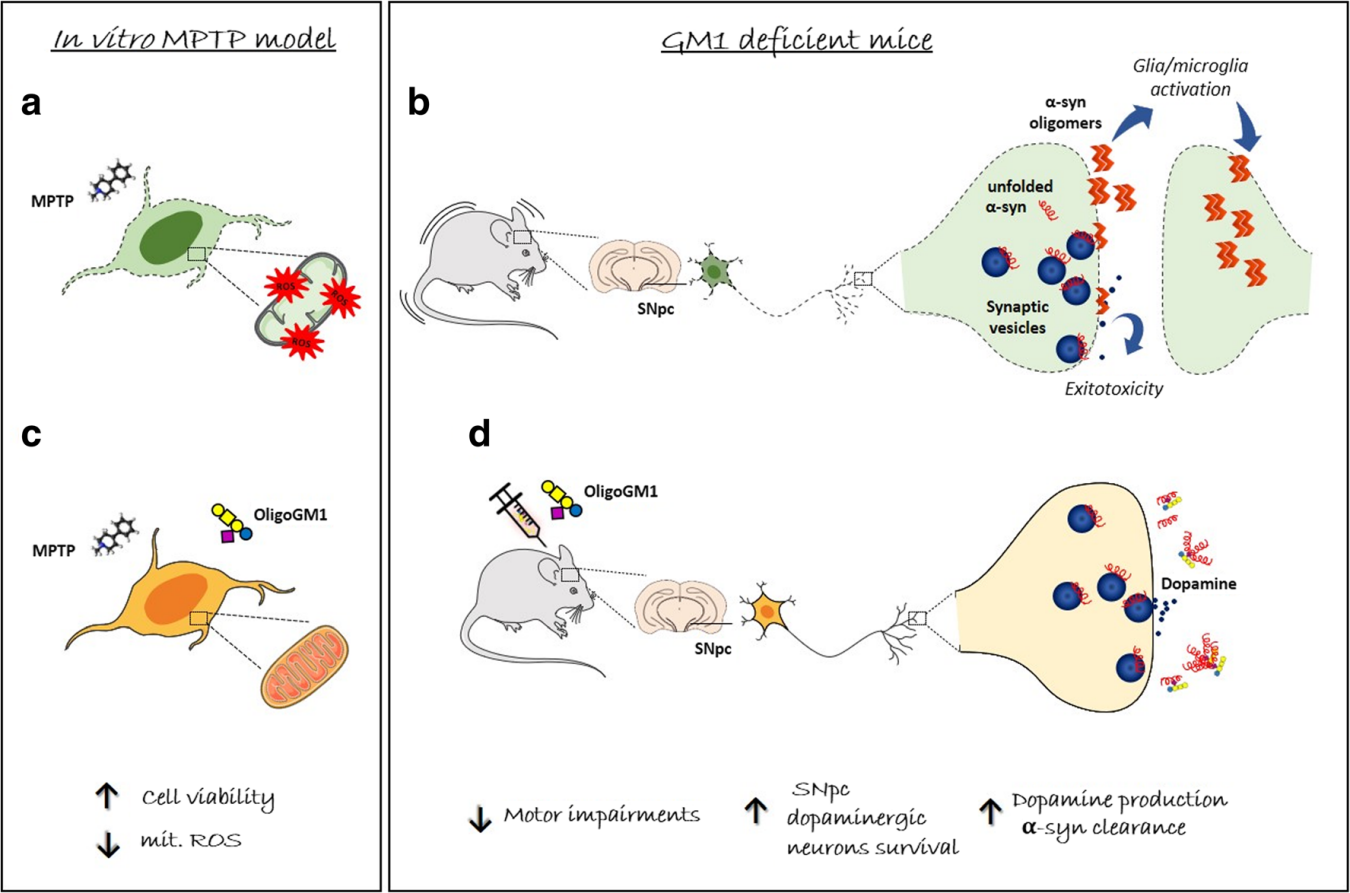
Schematic representation of OligoGM1-mediated neuroprotection in PD. On the left panel is shown the *in vitro* neuroprotective activity of OligoGM1 against MPTP neurotoxin, a widely accepted PD model. **a** MPTP exposure determines a deep mitochondrial impairment with a strong production of ROS that finally leads to cell death. **c** The administration of OligoGM1 strongly reduces the mitochondrial ROS content counteracting the MPTP toxicity and enhancing the cell survival [29]. On the right panel is represented the OligoGM1 capability to recover the PD features of GM1 deficient (*B4galnt1*^*+/−*^*)* mice. **b** The reduced GM1 content triggers a profound motor impairment due to the loss of substantia nigra pars compacta (SNpc) dopaminergic neurons lesioned by α-syn oligomers accumulation and to the consequent dopamine content reduction. **d** The chronic injection of OligoGM1 is able to completely counteract the PD phenotype: recovery of motor symptoms, rescue of dopaminergic neurons, dopamine levels and clearance of α-syn aggregates [94]. Sugar representation is according to [36]

A confirmation on this above came from the results obtained after daily intraperitoneal administration of OligoGM1 to *B4GalNT1*^*+/-*^ mice for 28 days [94]. Following the treatment, a complete recovery of motor skills and neurological lesions was observed, turning PD-animals to be comparable to the healthy wild-type ones. This *in vivo* results, together with the previous *in vitro* ones mentioned above, not only corroborates the importance of GM1 oligosaccharide chain as the essential and indispensable bioactive portion of the ganglioside, but it opens the way towards the therapeutic use of the carbohydrate in the context of PD (Fig. 7).

It would be interesting to evaluate the specific role of OligoGM1 in the aggregation and fibrillation of α-syn. It is known that the binding of α-syn to the surface of multilamellar vesicles loaded with GM1 favors the non-amyloidogenic form of the protein thus preventing its aggregation and pathogenicity [91, 95, 96]. The fact that OligoGM1 by itself can influence the conformation and pathogenicity of the protein could be a fascinating research line to investigate.

Replacement therapy with GM1 as a PD disease modifying agent has been attempted in clinical trials where the daily peripheral injection of 200 mg of GM1 for 120 weeks led, over a 2-year follow-up period, to a mild but significant slowdown of symptom progression [97]. Furthermore, a reduction trend in the decline in striatal dopamine transporter binding was found in the GM1 receiving group with respect to placebo counterpart [98]. The modest results obtained by this human trial, although performed by administering a very high dose of GM1 continuously, confirm that the potency of GM1 is severely limited by the fact that it crosses the BBB in very limited amount [11, 83, 94].

Given that the oligosaccharide chain of GM1 has completely different chemical-physical characteristics compared to the parental compound, a different behavior in the tissue distribution of OligoGM1 and, therefore, a diverse ability to reach the central nervous system could be expected. Although a complete pharmacokinetic study involving OligoGM1 has not yet been carried out, C57BL/6 mice intraperitoneally injected with OligoGM1 radiolabeled with tritium, isotopically incorporated into the external galactose, showed the presence of the pentasaccharide within the brain [94]. Any derivatives of OligoGM1 catabolism have not been identified, suggesting that following its peripheral administration the pentasaccharide remains intact for at least 24 hours [94]. These results show the ability of OligoGM1 to reach the central nervous system when injected peripherally and therefore its ability to somehow cross the BBB.

This study stimulated a subsequent research aimed at characterizing the transport of OligoGM1 through a human *in vitro* BBB model represented by human endothelial brain-like cells (hBLEC) grown and differentiated in a transwell system [99, 100]. The application of OligoGM1 and GM1 to the model highlighted the ability of OligoGM1 to cross the hBLEC layer with a 20-fold higher rate than that of GM1 [100]. Moreover, the permeability coefficients of OligoGM1 were comparable to those of equimolar quantities of Lucifer Yellow, a marker of paracellular passage (simple diffusion), differing only for the slightly larger size of the OligoGM1 molecule compared to Lucifer Yellow [100]. A paracellular transport for the saccharide was then easy to deduce, also supported by rigorous experiments showing that the OligoGM1 crossed the hBLEC even at 4°C, receiving only a modest slowdown that would have had to be almost total if its passage was due to ATP-dependent active transporters such as the ATP-binding cassette (ABC) family transporters [100]. The implication of an active mechanism in the OligoGM1 transport was elegantly excluded employing a naturally overexpressing ABC transporters cellular model, Caco2-cells [101], were no interaction of OligoGM1 with ABC protein family was found [100, 101].

Finally, it is important to note that the BBB transported OligoGM1 remained intact and able to induce neuritogenesis when administered to N2a neuroblastoma cells, indicating that it remained biologically active [100].

Certainly, the participation of other cellular species, such as glial cells, could influence the passage of OligoGM1 through the BBB and the achievement of target cells *in vivo*. Furthermore, as mentioned above, a detailed study on the mechanisms and kinetics of the sequestering of hematic OligoGM1 by the other organs and its tissue metabolism is necessary to define the best therapeutic dose. The experimentation of different routes of administration that better and earlier allow a cerebral localization of the saccharide (such as the intranasal one) is necessary as well.

## Concluding remarks

In a recent review, the ganglioside GM1 has been defined as a “*factotum* of nature” for its multitasking ability to regulate numerous processes of differentiation, functionality and maintenance of neuronal integrity. GM1 performs all these functions by interacting with many molecular partners that make it a “passepartout” for human life [11].

Humans are born with barely detectable level of complex gangliosides, comprising GM1, that slowly increase up to about the age of 20 and progressively decrease with aging [6]. After this phase a progressive decline is appreciated accompanied by cognitive decline. This process is governed by the temporary expression of specific glycosyltransferases that, working essentially in the Golgi apparatus, determine the pattern and quantity of each ganglioside [102]. The subjects who develop dementia prematurely together with the symptoms of neurodegeneration seem to present a reduced expression of some of these glycosyltransferases with a parallel reduction of the a-series gangliosides, i.e., of GM1 and its derivatives representing more complex structures [85, 87, 88].

From this evidence stems the theory on the sub-threshold reduction of the GM1 content as a risk factor for the development of neurodegenerative diseases such as Parkinson’s and Alzheimer’s diseases, at least for those forms still known today to have a sporadic origin [85, 87, 88, 94]. The proof of concept comes from preclinical data where GM1 replacement therapy reverses the neuropathological condition triggered by the GM1 deficit [83, 87, 90]. A further confirmation, at least partially, comes from clinical trials based on GM1 administration to Parkinson’s and Alzheimer’s patients, where modest benefits have been obtained [97, 98, 103, 104].

Although decades of preclinical and clinical research have highlighted the potential of GM1 for neuronal functioning, the mechanism of action behind it begins to become clearer only in recent years [9–11]. GM1 nature as membrane glycolipid has led to focus attention precisely on the cell glycocalyx, composed by the set of saccharide chains conjugated to membrane lipids and proteins that protrude from the lipid double layer in the extracellular environment. The importance of carbohydrates in recognition events has long been highlighted in cell adhesion, cell-cell recognition and cell-pathogen processes that are often regulated by the interactions of membrane carbohydrates with adjacent molecules. In this regard, in the last four years, the pentasaccharide portion of GM1 has been recognized as the bioactive component of this ganglioside, capable, even when isolated from the parental compound, to induce neuritogenesis and prevent neuronal toxicity at the same extent as GM1 was reported to do [28–33]. These cellular phenotypic effects are due to the carbohydrate interactions with membrane receptors, among which the neurotrophin’s receptor TrkA has been identified as a direct partner of the saccharide [28–33].

Following this event, a cascade of molecular pathway transducers is mobilized modulating cell motility, mitochondrial activity, immune response and regulation of intracellular calcium levels [29, 31, 33].

Therefore, it can be speculated that the abnormal decline in brain GM1 content associated with neurodegenerative diseases such as Parkinson’s results in the decrease in oligosaccharide chains in the neuronal glycocalyx and therefore in the insufficiency of their trophic support together with the aiding of the toxic amyloidogenic process of the α-syn protein. This new theory about the pathogenesis of PD gained support from the observations of reversion of the motor phenotype and of the neurological lesions obtained by the administration of OligoGM1 in a parkinsonian mouse model obtained by halving the endogenous GM1 levels [94].

In this way, the OligoGM1 not only represents a valid tool to explain the GM1 mechanism of action but, more importantly, it also represents an excellent therapeutic candidate against neurodegenerative diseases even more efficient than parental GM1 as its nature of small polar sugar gives it an advantage in reaching the central nervous system. Overall, despite the various gaps that still exist regarding OligoGM1 mechanism of action, the studies of this molecule represent an excellent example of the essentiality of fundamental science which favors the translational transfer of tools and approaches. Based on these premises, the possibility that OligoGM1 may soon reach a clinical trial phase to contrast the progression of PD seems to be concrete and based on solid pre-clinical evidence.

## Patent

A patent for “Oligosaccharides for the use in the treatment of Parkinson’s disease” has been registered by Milano University [105].

## Abbreviations

α-syn: α-synuclein
[^3^H]OligoGM1: tritium labeled GM1 oligosaccharide
[^3^H]OligoGM1-N_3_: tritium labeled photoactivable GM1 oligosaccharide
ABC: ATP-binding cassette family transporters
BBB: blood-brain barrier
DAG: diacyglycerol
ERK1/2: extracellular signal-regulated protein kinases 1 and 2
GM1: II^3^Neu5Ac-Gg_4_Cer, β-Gal-(1–3)-β-GalNAc-(1–4)-[α-Neu5Ac-(2–3)]-β-Gal-(1–4)-β-Glc-(1–1)-Cer
hBLEC: human endothelial brain-like cells
IP_3_: inositol 1,4,5-trisphosphate
MAPK: mitogen-activated protein kinase
MPP^+^: 1-methyl-4-phenylpyridinium
MPTP: 1-methyl-4-phenyl-1,2,3,6- tetrahydropyridine
N2a: Neuro2a cells
NGF: nerve growth factor
OligoGM1: GM1-oligosaccharide, II^3^Neu5Ac-Gg_4_, β-Gal-(1–3)-β-GalNAc-(1–4)-[α-Neu5Ac-(2–3)]-β-Gal-(1–4)-Glc
PD: Parkinson’s Disease
PKC: protein kinase C
p-ERK1/2: phosphorylated ERK1/2
p-TrkA: phosphorylated TrkA
ROS: reactive oxygen species
TrkA: neurotrophin tyrosin kinase receptor
Tyr490: tyrosine 490

## Declarations

The funders had no role in the design of the study; in the collection, analyses, or interpretation of data; in the writing of the manuscript, or in the decision to publish the results.
